# Editorial: WHO African region reforms: aligning country offices to strengthen health systems to meet global health objectives

**DOI:** 10.3389/fpubh.2025.1709533

**Published:** 2025-11-10

**Authors:** Obinna Onwujekwe, Joseph Chukwudi Okeibunor, Abdulmumini Usman, Olushayo Oluseun Olu

**Affiliations:** 1 Health Policy Research Group, Department of Pharmacology and Therapeutics, College of Medicine, University of Nigeria, Enugu, Nigeria; 2 World Health Organization Regional Office for Africa, Brazzaville, Republic of Congo

**Keywords:** transformation agenda, functional review, WHO, African region, Country Offices

## Introduction

The attainment of the global health development and humanitarian goals such as the Sustainable Development Goals (SDGs), Universal Health Coverage (UHC), and the Sendai Framework for Disaster Risk Reduction (SFDRR) amongst others require innovative approaches, organizational reforms and efficient management of resources to effectively address existing and emerging public health problems (1, 2). Furthermore, it creates opportunities for global and regional health institutions involved in shaping such agendas to strengthen their internal capacities to deliver cutting-edge technical assistance to their Member States (MS) and stakeholders. In Africa, weak health systems, fragile institutions, recurrent public health emergencies, and limited access to the social determinants of health highlight the urgent need for stronger regional public health capacity to support African countries to address the legitimate health needs of their populations (3). The unprecedented Ebola Virus Disease (EVD) outbreaks in West Africa (2014–2015) and the COVID-19 pandemic further exposed critical weaknesses in health system capacity (4, 5). These events underscore the urgent need to realign and strengthen both national and regional capacities in order to build resilient health systems.

Against the backdrop of the foregoing and growing concerns from stakeholders about the ability of the African Regional Office of the World Health Organization (WHO/AFRO) to adequately fulfill its normative functions (6), Dr Moeti, Regional Director (RD) of the WHO African Region (2015–2025) embarked on internal institutional reforms known as the Transformation Agenda (TA) at the inception of her term of office in February 2015 (7). The aim was to reposition the organization to support its MS in their drive to achieve the global health development and humanitarian goals. The goal of the TA was to enhance the health of the citizens of the WHO African Region (WHO/AFR).

The TA was an opportunity to improve internal management and capability of the WHO/AFRO and its 47 Country Offices (CO) for strengthening national health systems and enhancing global health security in Africa. As part of the TA, WHO/AFR undertook a Functional Review (FR) of all its 47 CO, which identified and realigned its workforce, plans and systems to the public health priorities of its MS (8). The FR which, was conducted from 2017 to 2019, and its results were implemented from 2019 to 2023,^1^ also generated evidence for context-specific strengthening of all 47 CO in line with the needs and priorities of each country; and introducing new ways of doing business.

The FR provided evidence on the usefulness of the TA and how the agenda can be improved on and sustained for the benefit of the WHO/AFR and its MS. The evidence and lessons learnt are important elements of evidence-based management (EBM) that is focused on “helping people, teams and organizations make better-informed decisions to help them achieve their goals by using intentional experimentation and feedback” (9).

Given the importance of the TA and FR as one of the key flagships and legacy projects of Dr. Moeti, it is important to translate the numerous opportunities, challenges and lessons learnt during the exercise into a scientific body of knowledge, especially within the framework of EBM. As articulated in the TA and FR, both frameworks were based on the underlying understanding that management decisions are optimally hinged on the best available evidence and reflections on the best way forward based on individual experiences.

The outputs from the various components of the assessments and realignment exercises were distilled into scientific articles that will inform a wider audience and hopefully lead to the adaptation and use of TA and FR in other WHO regions and contexts. The lessons learnt are useful and provide practical guidance to other African or global organizations that wants to conduct similar organizational reforms.

## Highlights of published articles

This Research Topic showcases nine empirical papers that were generated from examining various aspects of the FR. The papers that cover research articles, policy briefs, community case studies and commentaries are timely, evidence-informed and relevant to both country- and global-level organizational reform agendas. The foci of the papers reflect the identified management, implementation challenges and solutions in each of locus of the FR. The empirical data were derived from the evaluation of the FR by the different teams that contributed to the design of the FR. The papers cover a wide range of topics, within the ambit of FR.

To kick off, Petu et al. wrote an inspiring commentary titled “Consolidating the gains and alleviating the pains of the World Health Organization Africa Region Workforce Reform: a viewpoint.” The article showed that the FR produced several positive results within WHO/AFR's CO and that its benefits should continue to be nurtured to full realization and sustained. The article emphasized that this should be done as a long-term developmental framework that empowers the organization and its workforce to achieve its strategic goals effectively.

Langat et al. using a narrative synthesis approach, examined the literature to trace the temporal and contextual patterns underpinning Africa's pursuit of “health for all,” culminating in the ongoing drive toward UHC. They concluded that African countries have followed comparable contextual and chronological trajectories in implementing health reforms aimed at achieving healthcare for all, with a predominant focus on revitalizing primary health care (PHC). They recommended sustained investment in strong PHC infrastructure as indispensable, even as many countries adopt complex health insurance schemes as their principal UHC pathway.

In a case study titled, “Transforming technical assistance for more effective health services delivery in Africa: the WHO multi-country assignment teams experience” Ba et al. assessed the effectiveness of an innovative public health technical assistance delivery model called the Multi-Country Assignment Teams (MCATs). The MCATs, which was a product of the TA and FR aimed to provide quality and timely technical assistance to WHO/AFR MS. The case study showed that although several challenges persist that hinder the model's accelerated implementation, it is crucial to build on the successes so far achieved by the MCATs while addressing the challenges, particularly by improving awareness of their functions and ensuring adequate staffing and funding.

Ogundiran et al. conducted an evaluation of the Situations of Concern (SOC) classification system, developed by WHO/AFRO within the framework of the TA to assess and monitor COVID-19 epidemiological risk across the 47 MS of the Region. The review demonstrated that the SOC classifications informed timely operational decision-making in more than 70% of documented support cases. Moreover, the findings indicated that the application of SOC assessments facilitated the efficient management of limited resources and promoted a more equitable distribution of COVID-19 response support across affected communities.

Taderera examined the health policy lessons derived from the implementation of the TA and their potential contribution to strengthening health systems governance in Africa. The study found that training and mentorship enhanced the competencies of health leaders, thereby improving the effectiveness of TA outcomes. Additionally, the findings highlighted that collaboration with governance partners across all levels expanded the resource base for the TA while fostering stronger partnerships and improved communication. The author recommended sustained investment in leadership training as a means of reinforcing health systems governance to better meet population health needs.

Rwamatwara et al. documented the challenges associated with managing the workforce during the FR and its implementation process. Amongst other things, they outlined the processes undertaken to consolidate the human resources capacities of CO to actualize the aspirations of the FR. They described the challenges, good practices and lessons learned in managing the workforce during a period of uncertainty due to a robust reform of the magnitude of the FR. They concluded that major organizational reforms carry challenges such as uncertainty and anxiety among the workforce which should be carefully managed.

Usman, Cabore, et al. analyzed experiences and lessons from the FR process to provide insights, policy options, and recommendations for future reforms. Their findings indicated that stakeholder expectations were broadly consistent with WHO's mandate; however, meeting these expectations required additional technical expertise, increased staffing, and higher operational costs. Despite stakeholder support, limited donor funding necessitated modifications to implementation strategies. The study further highlighted that large-scale organizational reforms are both time- and resource-intensive, underscoring the need for clear communication and sustained commitment. The authors concluded that WHO/AFRO should prioritize the full implementation of FR outcomes to strengthen its capacity for evidence-based policy guidance, coordination, and high-level technical support at the country level.

In another paper, Usman, Olu, et al. evaluated health sector coordination mechanisms across the 47 Member States of the WHO African Region, with emphasis on WHO's role in facilitating coordination. The study identified generally weak and ineffective coordination platforms, often fragmented and programme-specific, and frequently characterized by parallel humanitarian and development systems with limited linkage to overarching frameworks. The authors emphasized the need to strengthen national coordination capacities and to clearly define WHO's coordination role in line with stakeholder expectations. They recommended context-specific approaches by CO, tailoring coordination mechanisms, functions, and tools to national circumstances to improve effectiveness.

Olu et al. assessed stakeholders' perceptions, expectations, and recommendations regarding WHO/AFR CO through a study conducted between 2017 and 2020. Findings indicated that while stakeholders expressed generally positive views of the CO, several negative perceptions persisted, potentially undermining the organization's credibility and effectiveness. The authors recommended a comprehensive reform programme to consolidate positive findings and address identified shortcomings. Key priorities include strengthening communication on WHO's roles and public health information, as well as establishing structured frameworks for engaging donors. Such efforts are essential to mitigate negative perceptions and enhance resource mobilization for health initiatives.

## Conclusion

Overall, the evidence presented in this Research Topic demonstrates that the FR has yielded several positive outcomes within WHO/AFR and its CO. These benefits should be further consolidated and sustained, particularly as part of a long-term developmental framework to strengthen the organization and its workforce in achieving its strategic objectives. The contributions in this Research Topic are expected to stimulate broader policy dialogue and encourage further research on health systems reforms among public health policymakers and researchers in Africa.

## Footnotes

1 The massive implementation of FR outcome was officially concluded in June 2023, however, WCOs continue to fill vacant positions as and when resources are mobilized.
